# Genetic network properties of the human cortex based on regional thickness and surface area measures

**DOI:** 10.3389/fnhum.2015.00440

**Published:** 2015-08-20

**Authors:** Anna R. Docherty, Chelsea K. Sawyers, Matthew S. Panizzon, Michael C. Neale, Lisa T. Eyler, Christine Fennema-Notestine, Carol E. Franz, Chi-Hua Chen, Linda K. McEvoy, Brad Verhulst, Ming T. Tsuang, William S. Kremen

**Affiliations:** ^1^Virginia Institute for Psychiatric and Behavioral Genetics, Department of Psychiatry, Virginia Commonwealth UniversityRichmond, VA, USA; ^2^Department of Psychiatry, University of California, San DiegoSan Diego, CA, USA; ^3^Center for Behavioral Genomics Twin Research Laboratory, University of California, San DiegoSan Diego, CA, USA; ^4^Mental Illness Research Education and Clinical Center, VA San Diego Healthcare SystemSan Diego, CA, USA; ^5^Department of Radiology, University of California, San DiegoSan Diego, CA, USA; ^6^Center of Excellence for Stress and Mental Health, VA San Diego Healthcare SystemSan Diego, CA, USA; ^7^Department of Neurosciences, University of California, San DiegoSan Diego, CA, USA

**Keywords:** imaging, genetic, twin, cortical thickness, surface area, network, small world, structural

## Abstract

We examined network properties of genetic covariance between average cortical thickness (CT) and surface area (SA) within genetically-identified cortical parcellations that we previously derived from human cortical genetic maps using vertex-wise fuzzy clustering analysis with high spatial resolution. There were 24 hierarchical parcellations based on vertex-wise CT and 24 based on vertex-wise SA expansion/contraction; in both cases the 12 parcellations per hemisphere were largely symmetrical. We utilized three techniques—biometrical genetic modeling, cluster analysis, and graph theory—to examine genetic relationships and network properties within and between the 48 parcellation measures. Biometrical modeling indicated significant shared genetic covariance between size of several of the genetic parcellations. Cluster analysis suggested small distinct groupings of genetic covariance; networks highlighted several significant negative and positive genetic correlations between bilateral parcellations. Graph theoretical analysis suggested that small world, but not rich club, network properties may characterize the genetic relationships between these regional size measures. These findings suggest that cortical genetic parcellations exhibit short characteristic path lengths across a broad network of connections. This property may be protective against network failure. In contrast, previous research with structural data has observed strong rich club properties with tightly interconnected hub networks. Future studies of these genetic networks might provide powerful phenotypes for genetic studies of normal and pathological brain development, aging, and function.

## Introduction

Discovery of organizational principles within the structure of human cortex will advance understanding of normal and abnormal behavior. Gene expression findings (reviewed by Dahmann et al., 2011) and human imaging twin studies (e.g., Schmitt et al., 2008) highlight the importance of genetics in determining structural patterning. We previously found orderly spatial patterns of genetic relationships between measures of brain size paralleling those seen in mouse cortex and derived two genetically-informed brain atlases based on cortical surface area (SA) (Chen et al., 2011) and cortical thickness (CT), respectively (Chen et al., 2013). (See Section Post-Processing and Image Analysis in Materials and Methods for an explanation of SA and CT parcellations.) Higher-level patterns of organization among measures of the size of these genetically-derived parcellations remain to be discovered. Previous studies have primarily examined correlations between structural measures in groups of unrelated individuals and within regions of interest based on cytoarchitecture such as Brodmann areas, sulcal/gyral boundaries, or on functional or connectivity data. Calculating regional size measures such as SA and mean CT for our genetically-derived parcellations, and examining patterns of association within a twin sample, could advance upon previous studies and improve our understanding of higher order patterning of brain structure.

Two main organizational patterns might be observed when examining genetic correlation between cortical size measures, and graph theory analysis has been used to discover properties of brain structural networks. For example, graph theory analysis applied to between-subject correlation matrices of regional size measures has indicated “small world” properties (He et al., 2007). Networks with small world properties are characterized by a combination of dense local interconnectivity (i.e., clustering) and short average path lengths between nodes (Watts and Strogatz, 1998). An example of a small world network is the *C. elegans* neural network, in which path lengths are small and the number of links required to connect the network is minimal, leading to increased efficiency. Only one other study has examined network properties emerging from genetic correlation matrices between structural measures within human anatomical brain regions (Schmitt et al., 2008). In this study of correlations among cortical thickness measures within sulcal/gyral regions in children and adolescents, small world properties were observed.

Another organizational principle that has been observed for brain size data is that of a “rich club network,” characterized by a highly integrated, connected group of high-level nodes with the ability to easily transfer to several other nodes. This organization protects the network from critical failure, as any rich node can easily distribute to several other nodes. Power gradients tend to have strong rich club properties, as hub stations can easily distribute to many others to avoid failure of the system (van den Heuvel and Sporns, 2011). An example of an absence of such rich club properties might be neurons within a sparse coding network, or modalities within the visual cortex, where each node bears a relatively unique and irreplaceable function (Lettvin et al., 1959). Previous studies have observed rich club network properties within brain structural data (van den Heuvel and Sporns, 2011; van den Heuvel et al., 2013; Collin et al., 2014) but rich club properties have not been examined with respect to genetic associations or using size measures within genetically-defined parcellations as nodes of interest.

In the present study, we explored the genetic relationships among SA and CT measures within genetically-informed cortical parcellations using three techniques: biometrical genetic modeling, cluster analysis, and graph theoretical models. We show that shared genetic covariance between regional CT and SA calculated in this way may exhibit small world, but not rich club, network properties. These genetic network models may serve to complement existing network models of anatomical and functional relationships within the human connectome.

## Materials and methods

### Participants

Data were obtained as part of the Vietnam Era Twin Study of Aging (VETSA), a longitudinal study of cognitive and brain aging with baseline in midlife (Kremen et al., 2006, 2013). Participants in VETSA were sampled from the Vietnam Era Twin (VET) Registry, a nationally distributed sample of male-male twin pairs, who served in the United States military at some point between 1965 and 1975 (Goldberg et al., 2002). Detailed descriptions of the VET Registry's composition and method of ascertainment have been reported by Eisen et al. (1989) and Henderson et al. (1990). Men (*N* = 1237) aged 51–60 participated in the primary VETSA project, with a mean age = 55.4 years (*SD* = 2.5). Participants were predominantly Caucasian (89.7%), with an average education of 13.8 years (*SD* = 2.1). In comparison to U.S. census data, participants in the VETSA are similar in health and lifestyle characteristics to American men in their age range (Schoeneborn and Heyman, 2009). In order to be eligible for VETSA, both members of a twin pair had to agree to participate and be between the ages of 51 and 59 at the time of recruitment. We determined zygosity for 92% of the sample by 25 microsatellite markers obtained from blood; zygosity for the remainder was determined with combined questionnaire and blood group methods. Past comparison of the two approaches in the VETSA sample has demonstrated agreement of 95%. Eligible participants were recruited to participate in the MRI portion of the study. Data from the cortical parcellations derived from the cluster analyses (imaging methods described below) were available on 429 of the VETSA participants (100 MZ twin pairs, 70 DZ twin pairs, and 89 unpaired twins).

Participants chose either the University of California San Diego (UCSD) or Boston University for a day-long protocol of medical, physical, psychosocial, and neurocognitive assessments. Informed consent was obtained from all participants prior to data collection, the data collection was approved by the relevant institutional review boards, and all research conformed to relevant regulatory standards. Standard MRI exclusion criteria were imposed in order to ensure participant safety (e.g., the absence of metal in the body).

### Imaging acquisition

Neuroimaging was performed at either UCSD or the Massachusetts General Hospital (MGH) in Boston. All images were acquired on Siemens 1.5 Tesla scanners. Sagittal T1-weighted MPRAGE sequences were employed with a TI = 1000 ms, TE = 3.31 ms, TR = 2730 ms, a flip angle = 7°, slice thickness = 1.33 mm, and voxel size of 1.3 × 1.0 × 1.3 mm. Raw DICOM MRI scans from both sites were transferred to MGH for initial post-processing and quality control, and then to UCSD for further image analysis.

### Post-processing and image analysis

SA expansion and CT mapping were conducted using methods based on the publicly available FreeSurfer software package (Fischl et al., 2002). The end result was a measure, at each vertex (a very small triangle) on the surface of the brain (with < 1 mm mean vertex spacing), of both CT and SA. CT is the distance of a line drawn between pial and white matter surfaces perpendicular to the white matter at this point, and SA is the area of each triangle that has to be expanded or contracted to fit a template.

We then calculated CT and SA within genetically-derived parcellations of the cortical mantle; derivation of the boundaries of these parcellations is described in Chen et al. (2012, 2013). Briefly, fuzzy cluster analysis was applied to the genetic correlations between SA-values at each vertex on the cortical surface and, separately, to the genetic correlations between CT-values at each vertex on the cortical surface. CT measures were adjusted for age, scanner and mean CT, and SA measures were adjusted for age, scanner, and total SA. Based on a silhouette coefficient, 12 parcellations per hemisphere were determined to be optimal for both CT and SA, with the parcellation boundaries being very similar for left and right hemispheres. Thus, the boundaries of each CT and SA parcellation used in the present study are based on patterns of genetic correlation among thickness or surface area values, respectively, at each vertex. The boundaries of the parcellations were quite similar for CT and SA, though not identical (see Chen et al., 2013 for discussion of differences). Regions are named for the general vertex-wise cluster location, but the degree to which SA and CT homologous regions overlap on the cortical surface varies depending on the structure. Thus, for the present analysis, CT parcellation boundaries were applied to the vertex-based CT measures in order to calculate a mean CT within each parcellation, and SA parcellation boundaries were applied to vertex-based SA measures to calculate the SA of each region. Thus, our process is analogous to more traditional analyses that might use cytoarchitectonic or physical features to carve up the cortex into mutually-exclusive regions and then calculate the surface area and mean thickness within each of the anatomically-based regions. In contrast, our boundaries were determined by examining shared genetic influences across the cortex, which should lead to more genetically-homogenous regions over which to average (mean CT) or sum (SA) for our present analysis of regional genetic associations. We have given each of these regions a name based on the approximate anatomical location (Table 1) and basically homologous regions in the CT and SA parcellation schemes were given the same name.

**Table 1:** **Parcellation surface area and cortical thickness**.

**Parcellation**	**Abbreviation**
1. Motor–Premotor (Central)	C
2. Occipital	O
3. Postlateral temporal	PLT
4. Superior parietal	SP
5. Orbitofrontal	OF or F
6. Superior temporal	ST
7. Inferior parietal	IP
8. Dorsomedial frontal	DMF
9. Anteromedial temporal	AMT or A
10. Precuneus	PRC
11. Dorsolateral prefrontal	DLP
12. Pars opercularis	PRS

*Abbreviations in **Figures 2, 3, 6, 7** and Mean Nodal Degree*.

### Statistical analyses

Structural equation modeling was used to examine genetic contributions to individual differences in global-adjusted CT and SA across the 48 parcellations (24 regional CT measures and 24 regional SA measures) using a standard model for bivariate twin data. Details of biometric modeling procedures are described below. Maximum likelihood-based genetic analysis of CT and SA of all 48 parcellations in one multivariate model was not practical due to sample size. Instead, we conducted a combination of univariate (*n* = 48) and all possible bivariate analyses of the entire set of parcellations (*n* = 1128). Cluster and graph theory analyses were employed to investigate the network properties of the genetic covariance matrices.

#### Univariate ACE analyses

The parcellation-based CT and SA regional measures were analyzed using OpenMx (Boker et al., 2010), a package for structural equation and other statistical modeling in the R language (R Development Core Team, 2008). Model fitting was conducted using full-information maximum likelihood. This likelihood-based approach allows tests of the statistical significance of parameters of interest from the original model, by fixing them to pre-specified values to form a sub-model.

In the classical univariate twin design, the variance of a phenotype is decomposed into the proportion of total variance attributed to additive genetic (A) influences, common or shared environmental (C) influences, and unique environmental (E) influences (Eaves et al., 1978; Neale and Cardon, 1992). The name “ACE model” is derived from these three components of variance. Additive genetic influences are assumed to correlate 1.0 between MZ twins who generally share 100% of their genes, and 0.5 between DZ twins who on average share 50% of their segregating genes. Shared environment is assumed to correlate 1.0 between both members of a twin pair, regardless of twin zygosity. Unique environmental influences are assumed to be uncorrelated between the members of a twin pair. Measurement error is also included in the E term because it is also assumed to be uncorrelated between twins.

The proportion of a phenotype's total variance attributable to additive genetic influences is considered the heritability of the phenotype. The significance of genetic or shared environmental influences was tested by fixing the parameter in question to zero, and then comparing the fit of the reduced model against the full model. For each of the 48 variables, we tested the significance of change in model fit when genetic, shared environmental or unique environmental variance components were removed.

Under certain regularity conditions, the difference in twice negative log-likelihood (-2lnL) asymptotically follows a χ^2^ distribution, with degrees of freedom equal to the difference in the number of free parameters in the two models. Variance components such as *a*^2^ and *c*^2^ do not meet these regularity conditions, due to a lower bound of zero (Dominicus et al., 2006; Wu and Neale, 2012). Under the null hypothesis, tests for variance components are distributed as a 50:50 mixture of χ^2^ with one degree of freedom and zero, and are corrected by simply halving the *p*-value.

#### Bivariate AE analyses

**Figure 1:**
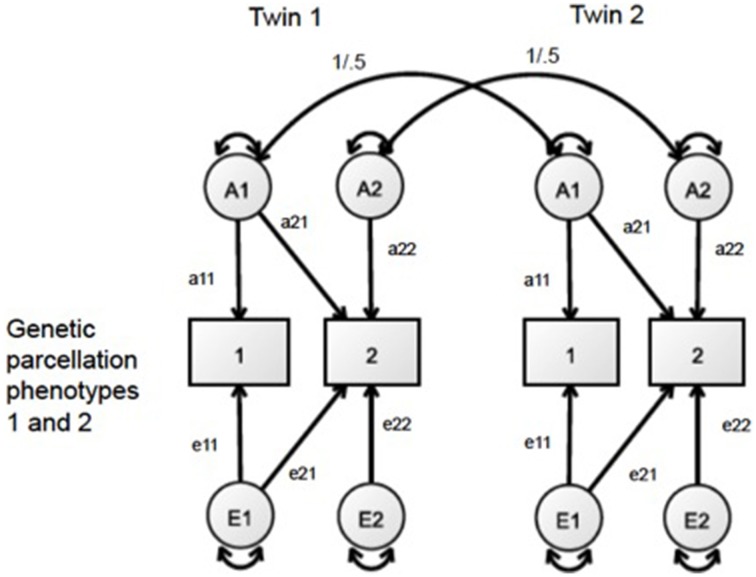
**Bivariate AE Cholesky framework, in which the covariance of two phenotypes is decomposed into genetic (A) and unique environmental (E) variance**.

To analyze the pattern of genetic relationships between the regional measures of SA and CT, we adopted a bivariate AE approach. The bivariate Cholesky decomposition of each pair of regional size measures partitions the covariance due to genetic (*a*^2^) and environmental (*e*^2^) origin. In this analysis, we left out a shared environmental variance component (*c*^2^) from the models due to a lack of any significant shared environmental variance across all univariate analyses of the regional measures. A simplified diagram of this bivariate Cholesky model is presented in Figure 1. The genetic correlation between every pair of parcellation-based CT and SA measures was calculated by standardizing the shared genetic covariance between each pair of parcellation size measures. Analyses of all 1128 pairwise models populated a 48 × 48 genetic correlation matrix. The precision of the genetic correlations varies due to the total amount of genetic variance, so these derived statistics are unsuitable summary statistics for confirmatory factor analysis. However, they are useful for cluster and graph theory analyses (Schmitt et al., 2008).

#### Cluster analysis

We then conducted exploratory analyses of the genetic correlation matrix using cluster analysis and graph theoretical modeling. The 48 × 48 genetic correlation matrix was visualized using the heatmap.2 function in the gplots package of R (Warnes et al., 2015). The heatmap.2 function performs hierarchical analysis using Euclidian distances and a stepwise clustering strategy to create a hierarchy of distance-based mapping. Genetic regions are spatially proximal in the matrix corresponding to the likeness of their correlational patterns.

#### Graph theory

Finally, we constructed alternate visualizations of the relationships between parcellation-based CT and SA using graph theoretical models. In our data, significant edges (lines) were defined by genetic correlations significant at α < 0.05. No correction for multiple testing was applied because these analyses were exploratory. We identified significant edges by comparing the fit of a bivariate Cholesky decomposition with a submodel in which the path allowing for genetic covariance was removed.

From this network graph, we calculated statistical properties of the network: characteristic path length (L) and the clustering coefficient (C) (Watts and Strogatz, 1998). Within the present data, values of L and C for the system as a whole were calculated by taking mean values for all nodes in the graph. We compared these calculations with those from 1000 simulated Watts–Strogatz networks, each with the same number of nodes and edges as the real data. In the simulations, for each of *i* edges, two nodes were identified by sampling from a pool of 48 nodes (with replacement) with uniform probability. Analysis and visualization of graphs was performed using Watts–Strogatz functions in the igraph 0.6.5 (Csardi, 2013) and the qgraph (Epskamp et al., 2012) packages for R.

We also examined the extent to which networks evidenced rich club properties. Rich club properties were operationalized using the *k* coefficient, as defined by

(1) $$\begin{array}{l} \phi k\;=\;\frac{{2E}_{>k}}{N_{>k}\left(N_{>k}-1\right)}, \end{array}$$

Where *E* is the number of connections between nodes greater than degree *k*, and *N* is the number of nodes greater than degree *k*. The high degree of interconnectedness among nodes of a high degree makes the rich club network distinctive and protects the network from critical failure, as any rich node can easily distribute to several other nodes. Across genetic parcellations, we derived the number of nodes exhibiting connections exceeding degree *k*, and calculated the ratio of twice the number of connections between the >*k* nodes relative to the maximum number of connections possible. A larger Φ*k* reflects rich club properties and suggests some integration of the rich nodes. If Φ*k* is found to be greater than 1, the larger likelihood of node connections as the number of nodes increases can then be managed by weighting Φ*k*:

(2) $$\begin{array}{l} \phi_{unc}\left(k\right)\underset{k,k_{{max}^{\to \infty }}}{\sim }\frac{k^{2}}{\langle k\rangle N}, \end{array}$$

where *k*_max_ is the maximum degree of any node. The subsequent calculation of ρ_unc_(*k*) = Φ*k*/Φ_unc_(*k*) provides a weighted ratio in which values >1 indicate the presence of rich club properties.

## Results

### Variance component analyses

Variance decomposition demonstrated substantial heritability of SA within a majority of genetically-derived SA parcellations (ranging from 0.34 for right superior temporal to 0.69 for left postlateral temporal; median = 0.53) and for mean CT within some genetically-derived CT parcellations (ranging from 0.0 for right inferior parietal to 0.55 for right occipital; median = 0.34). Tables 2, 3 present heritability estimates for the parcellation-based regional measures, as well as tests of the statistical significance of genetic and shared environmental effects. Genetic variance was more consistently substantial for SA in many regions, and the shared environment appeared to have no significant effect on observed variability in SA within the parcellations. Genetic variance was substantial for CT in some of the regions, and shared environment appeared to have no significant effect on observed inter-individual variability in parcellation-based thickness in all areas.

**Table 2:** **ML parameter estimates and ***p***-values from hypothesis testing of univariate ACE models of surface area**.

	**Variance components (lower; upper 95% CI)**						**Hypothesis test**		
							***p*-Values^a^**		
	***a***^**2**^		***c***^**2**^		***e***^**2**^		**A**	**C**	**AC**
**LEFT**									
Motor–Premotor	0.58	(0.30;0.69)	0.00	(0.00;0.23)	0.42	(0.31;0.55)	**0.00**	1.00	**0.00**
Occipital	0.38	(0.00;0.71)	0.24	(0.00;0.56)	0.38	(0.28;0.51)	0.05	0.23	**0.00**
Postlateral temporal	0.69	(0.43;0.77)	0.00	(0.00;0.23)	0.31	(0.23;0.42)	**0.00**	1.00	**0.00**
Superior parietal	0.53	(0.11;0.72)	0.09	(0.00;0.46)	0.38	(0.28;0.51)	**0.01**	0.67	**0.00**
Orbitofrontal	0.51	(0.24;0.63)	0.00	(0.00;0.22)	0.49	(0.37;0.64)	**0.00**	1.00	**0.00**
Superior temporal	0.49	(0.07;0.62)	0.00	(0.00;0.36)	0.51	(0.38;0.66)	**0.02**	1.00	**0.00**
Inferior parietal	0.40	(0.14;0.55)	0.00	(0.00;0.00)	0.60	(0.45;0.77)	**0.01**	1.00	**0.00**
Dorsomedial frontal	0.61	(0.32;0.71)	0.00	(0.00;0.25)	0.39	(0.29;0.52)	**0.00**	1.00	**0.00**
Anteromedial temporal	0.46	(0.10;0.59)	0.00	(0.00;0.31)	0.54	(0.41;0.69)	**0.02**	1.00	**0.00**
Precuneus	0.61	(0.33;0.71)	0.00	(0.00;0.24)	0.39	(0.29;0.51)	**0.00**	1.00	**0.00**
Dorsolateral prefrontal	0.65	(0.41;0.74)	0.00	(0.00;0.20)	0.35	(0.26;0.48)	**0.00**	1.00	**0.00**
Pars opercularis	0.57	(0.15;0.71)	0.04	(0.00;0.39)	0.39	(0.29;0.53)	**0.01**	0.84	**0.00**
**RIGHT**									
Motor–Premotor	0.57	(0.28;0.68)	0.00	(0.00;0.00)	0.43	(0.32;0.57)	**0.00**	1.00	**0.00**
Occipital	0.53	(0.09;0.64)	0.00	(0.00;0.38)	0.47	(0.36;0.61)	**0.02**	1.00	**0.00**
Postlateral temporal	0.51	(0.21;0.63)	0.00	(0.00;0.24)	0.49	(0.37;0.64)	**0.00**	1.00	**0.00**
Superior parietal	0.54	(0.32;0.66)	0.00	(0.00;0.17)	0.46	(0.34;0.61)	**0.00**	1.00	**0.00**
Orbitofrontal	0.53	(0.12;0.65)	0.00	(0.00;0.34)	0.47	(0.35;0.62)	**0.01**	1.00	**0.00**
Superior temporal	0.34	(0.00;0.58)	0.11	(0.00;0.47)	0.56	(0.42;0.74)	0.21	0.66	**0.00**
Inferior parietal	0.45	(0.21;0.59)	0.00	(0.00;0.17)	0.55	(0.41;0.73)	**0.00**	1.00	**0.00**
Dorsomedial frontal	0.62	(0.40;0.72)	0.00	(0.00;0.00)	0.38	(0.28;0.51)	**0.00**	1.00	**0.00**
Anteromedial temporal	0.50	(0.11;0.61)	0.00	(0.00;0.34)	0.50	(0.39;0.64)	**0.02**	1.00	**0.00**
Precuneus	0.42	(0.10;0.56)	0.00	(0.00;0.25)	0.58	(0.44;0.75)	**0.02**	1.00	**0.00**
Dorsolateral prefrontal	0.60	(0.16;0.73)	0.04	(0.00;0.43)	0.36	(0.27;0.49)	**0.01**	0.85	**0.00**
Pars opercularis	0.56	(0.25;0.67)	0.00	(0.00;0.26)	0.44	(0.33;0.58)	**0.00**	1.00	**0.00**

a *p-Values reflect tests of the hypotheses of no genetic effect on phenotypic variance (A), no shared environmental effect (C), and no familial (AC) effects. Statistically significant effects (α = 0.05) are shown in boldface*.

**Table 3:** **ML parameter estimates and ***p***-values from hypothesis testing of univariate ACE models of cortical thickness**.

	**Variance components (lower, upper 95% CI)**						**Hypothesis test**		
							***p*-Values^a^**		
	***a***^**2**^		***c***^**2**^		***e***^**2**^		**A**	**C**	**AC**
**LEFT**									
Motor–Premotor	0.21	(0.00;0.45)	0.09	(0.00;0.39)	0.70	(0.55;0.87)	0.47	0.72	**0.00**
Occipital	0.34	(0.00;0.66)	0.22	(0.00;0.56)	0.44	(0.33;0.59)	0.14	0.35	**0.00**
Postlateral temporal	0.26	(0.00;0.62)	0.25	(0.00;0.55)	0.49	(0.37;0.65)	0.27	0.30	**0.00**
Superior parietal	0.15	(0.00;0.58)	0.33	(0.00;0.55)	0.52	(0.40;0.66)	0.53	0.18	**0.00**
Orbitofrontal	0.46	(0.00;0.59)	0.00	(0.00;0.43)	0.54	(0.41;0.71)	0.09	1.00	**0.00**
Superior temporal	0.53	(0.08;0.65)	0.00	(0.00;0.39)	0.47	(0.35;0.61)	**0.02**	1.00	**0.00**
Inferior parietal	0.37	(0.00;0.51)	0.00	(0.00;0.34)	0.63	(0.49;0.79)	0.08	1.00	**0.00**
Dorsomedial frontal	0.42	(0.00;0.68)	0.16	(0.00;0.53)	0.42	(0.32;0.55)	0.06	0.51	**0.00**
Anteromedial temporal	0.52	(0.07;0.65)	0.03	(0.00;0.43)	0.45	(0.35;0.58)	**0.02**	0.88	**0.00**
Precuneus	0.09	(0.00;0.51)	0.31	(0.00;0.50)	0.60	(0.47;0.74)	0.74	0.22	**0.00**
Dorsolateral prefrontal	0.28	(0.00;0.57)	0.17	(0.00;0.51)	0.55	(0.43;0.70)	0.31	0.56	**0.00**
Pars opercularis	0.34	(0.00;0.59)	0.12	(0.00;0.48)	0.54	(0.41;0.69)	0.16	0.60	**0.00**
**RIGHT**									
Motor–Premotor	0.44	(0.00;0.57)	0.00	(0.00;0.40)	0.56	(0.43;0.72)	0.07	1.00	**0.00**
Occipital	0.41	(0.00;0.60)	0.07	(0.00;0.47)	0.52	(0.40;0.68)	0.10	0.78	**0.00**
Postlateral temporal	0.14	(0.00;0.49)	0.19	(0.00;0.43)	0.67	(0.51;0.84)	0.61	0.44	**0.00**
Superior parietal	0.33	(0.00;0.61)	0.17	(0.00;0.55)	0.50	(0.39;0.63)	0.20	0.54	**0.00**
Orbitofrontal	0.55	(0.30;0.67)	0.00	(0.00;0.18)	0.45	(0.33;0.61)	**0.00**	1.00	**0.00**
Superior temporal	0.20	(0.00;0.58)	0.25	(0.00;0.51)	0.55	(0.41;0.71)	0.40	0.23	**0.00**
Inferior parietal	0.00	(0.00;0.42)	0.32	(0.00;0.45)	0.68	(0.55;0.82)	1.00	0.16	**0.00**
Dorsomedial frontal	0.44	(0.09;0.56)	0.00	(0.00;0.29)	0.56	(0.44;0.72)	**0.02**	1.00	**0.00**
Anteromedial temporal	0.20	(0.00;0.55)	0.21	(0.00;0.48)	0.59	(0.45;0.75)	0.42	0.35	**0.00**
Precuneus	0.38	(0.00;0.52)	0.00	(0.00;0.34)	0.62	(0.48;0.78)	0.06	1.00	**0.00**
Dorsolateral prefrontal	0.53	(0.27;0.65)	0.00	(0.00;0.20)	0.47	(0.35;0.62)	**0.00**	1.00	**0.00**
Pars opercularis	0.17	(0.00;0.56)	0.26	(0.00;0.51)	0.56	(0.43;0.72)	0.48	0.25	**0.00**

a *p-Values reflect tests of the hypotheses of no genetic effect on phenotypic variance (A), no shared environmental effect (C), and no familial (AC) effects. Statistically significant effects (α = 0.05) are shown in boldface*.

### Bivariate genetic analyses

**Figure 2:**
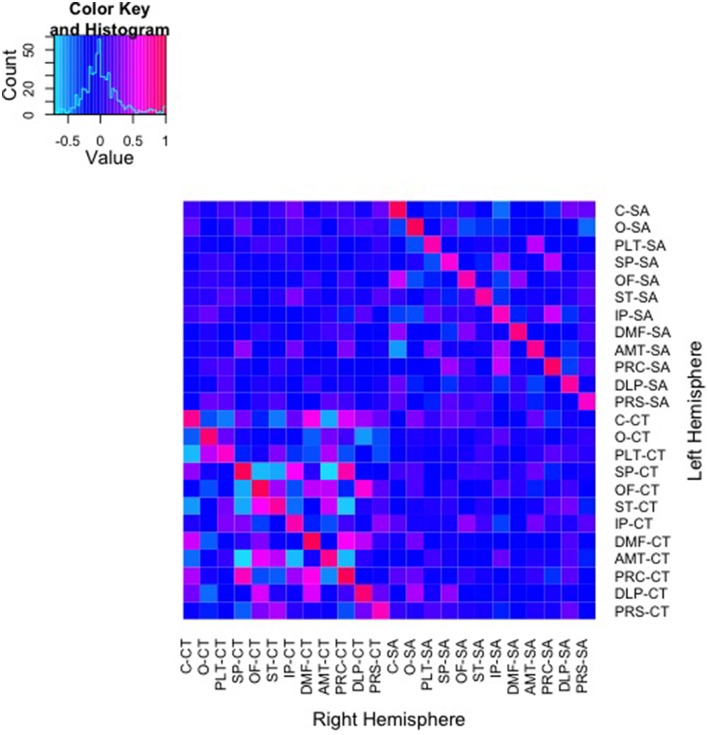
**Heatmap of genetic correlations between 48 bivariate bilateral genetic SA and CT parcellations**. Corresponding left (L) and right (R) structures are adjacent. The color scale represents the weighted genetic correlations within and between parcellations.

Color mapping of the genetic correlation matrix is shown in Figure 2. In these analyses, shared environmental variance (*c*^2^) was excluded from the models due to a lack of any significant shared environmental variance across all univariate analyses of the regional measures. Genetic correlations derived from AE models between SA of the parcellations ranged from −0.56 to 1.0. Genetic correlations between parcellation-based mean CT ranged from −0.71 to 1.0. As can be seen on the diagonal of the heatmaps, bilaterally homologous regions were more likely to be positively genetically correlated.

### Cluster analysis

**Figure 3:**
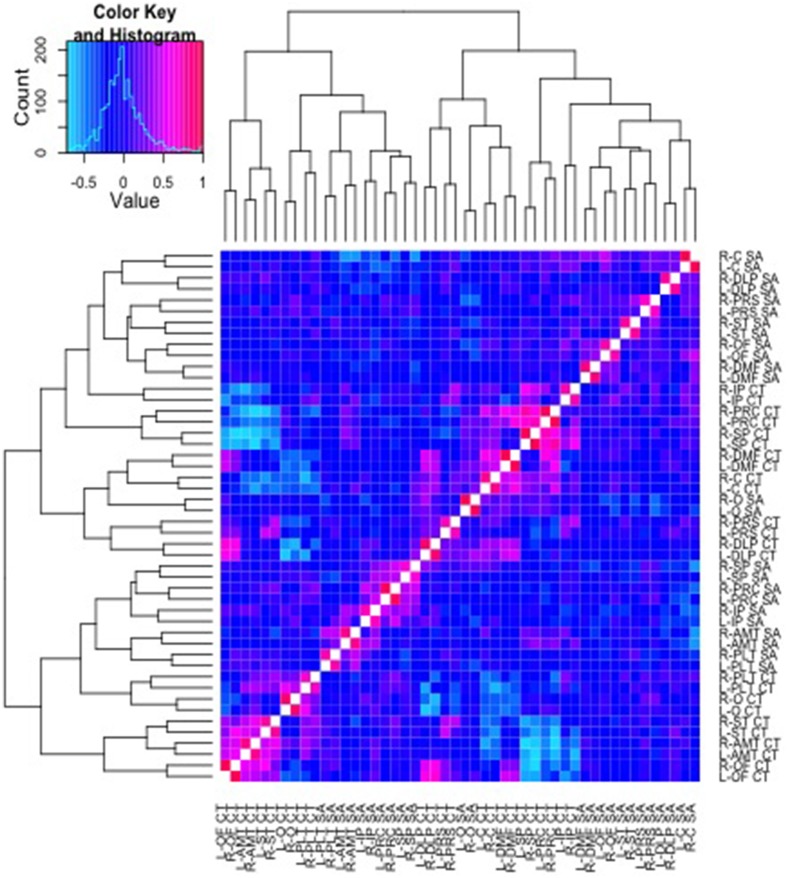
**Cluster analysis of the genetic correlations and respective dendrogram for all 48 parcellation phenotypes combined**. Heatmap2.0 for R includes specifications allowing the order of the parcellations to vary along the x and y axis, according to the strength of genetic relationship between parcellations. Here, a corresponding dendrogram is depicted along the left and upper edges of the diagram, indicating the observed genetic structure of the cluster groups.

Pictured in Figure 3 are results from a cluster analysis with a dendrogram depicting similarity in the patterns of genetic correlations across both SA and CT parcellation values. As can be observed in the figure, the CT and SA parcellation values tend to cluster together in small groups, though the clusters are not entirely divided based on the type of measure (CT or SA).

### Graph theoretical properties

Using genetic correlations with *p* < 0.05 as a threshold for edge placement, we used only the lower half of the matrix and identified 222 unique edges connecting the 48 parcellation-based measures of CT and SA. We used binarized edges for the network analyses resulting in undirected, non-weighted graphs. The mean degree of the network (4.63) was greater than the natural log of the number of nodes (3.87), thus small world properties were estimable (Watts and Strogatz, 1998; Achard et al., 2006). We modeled the distribution of the clustering coefficient for 1000 Watts–Strogatz networks generated using permutations of observed (unweighted) data, and the observed data fell within the Watts–Strogatz network distribution at 0.10 for the 48 nodes, and 0.24 and 0.08 for CT and SA, respectively. Average path lengths were 0.08, 0.11, and 0.17, for all 48 nodes and for CT and SA, respectively. Sigma, defined as the ratio of the clustering coefficient to the characteristic path length, was 1.25 for the 48 nodes. The quantity of significant genetic correlations is comparable in this sample relative to what would be expected from a small world network.

**Figure 4:**
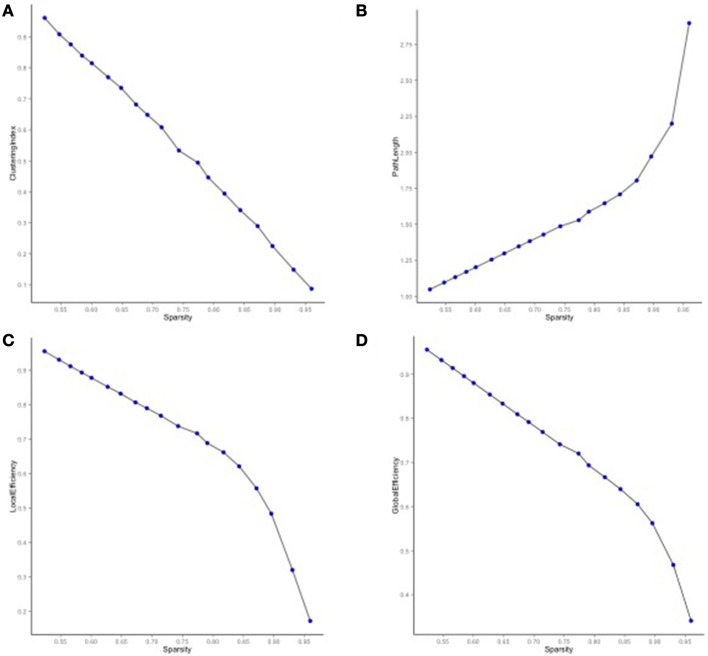
**Network properties for SA and CT using different ***p***-value thresholds as a function of sparsity degree**. **(A)** The clustering index decreased as sparsity degree increased. **(B)** The characteristic path length increased as sparsity degree increased. **(C)** Local and **(D)** global efficiency decreased as sparsity increased.

**Figure 5:**
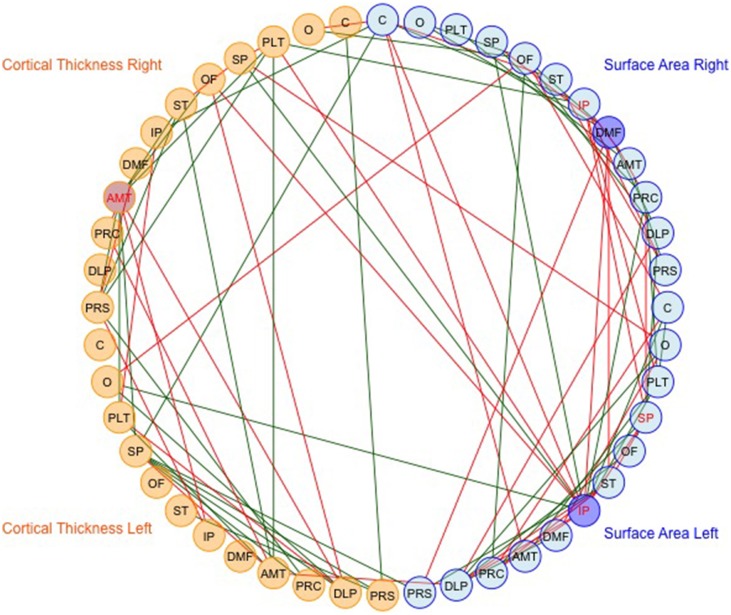
**The observed data for SA and CT of the parcellations combined**. Table 1 presents the parcellation abbreviations for reference. Edges represent genetic correlations where *p* < 0.05. Red edges denote negative genetic correlations, and green edges denote positive genetic correlations. The right side of the diagram depicts SA cluster nodes (in blue) and the left side depicts CT cluster nodes (in orange). Darker shaded circles denote parcellations with the highest nodal degree within SA or CT, and red lettering denotes parcellations with highest nodal degree across all 48 parcellations. The bilaterally symmetrical CT correlations are sparser than bilateral SA correlations.

We then mapped edges as they were observed in the data. Table 1 presents the parcellation abbreviations for reference. Sparsity of the binarized SA and CT correlation matrices was 0.109 and 0.174, respectively, using a standard sparsity degree. Figure 4 presents changes in network attributes as a function of sparsity degree across different *p*-value thresholds. A radial convergence diagram, Figure 5, depicts both SA and CT regional measures combined, with SA on the right and CT on the left. In this figure, associations of *p* < 0.05 were mapped with the positive associations in green and negative associations in red. Sparsity of the binarized lower triangular matrix of genetic correlations *p* < 0.05 for all 48 nodes was 0.197. The lower triangular matrix of genetic correlation values with only non-significant *p*-values exhibited a sparsity of 0.803.

**Figure 6:**
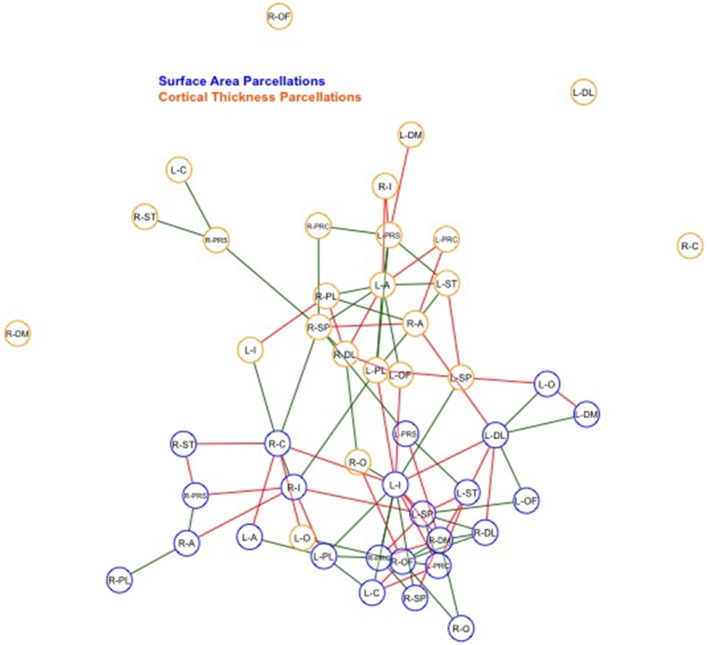
**Above is a final network representation of genetic correlations where ***p*** < 0.05, and where the proximity of the edges denotes strength of genetic correlation between parcellations**. Table 1 presents parcellation abbreviations for reference. Red edges reflect negative genetic correlations, and green edges reflect positive genetic correlations. SA parcellations are outlined in blue and CT cluster nodes are outlined in orange. R- and L-prefixes denote right and left hemispheric parcellations. Note increased edges stemming from right frontal SA, right central SA, and left medial CT. Two distinct local networks appear to surround the right frontal SA and left medial CT parcellations.

**Figure 7:**
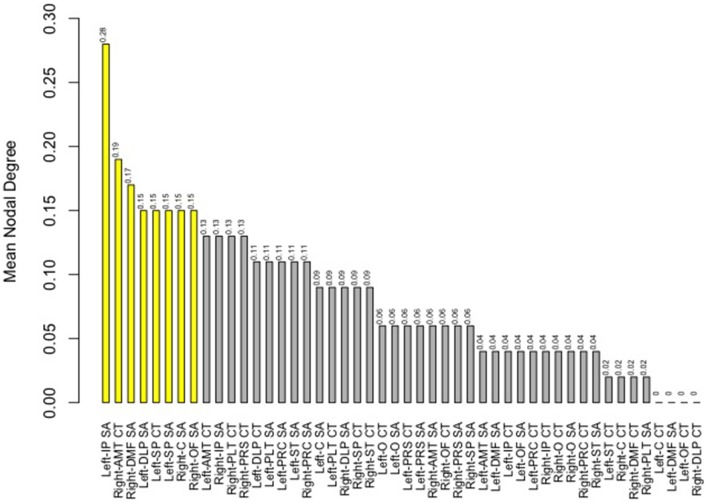
**Above is a barplot of the average nodal degree of each of the parcellations, from greatest to least**. Table 1 presents the parcellation abbreviations for reference. Yellow bars indicate parcellations >1.0 standard deviation from the mean.

Finally, we examined strength of genetic association via the spatial proximity of nodes. Note that this does not refer to spatial proximity of the cortical parcellations on the surface of the brain, but rather how “close” they are in the network, which depends of the strength and pattern of the genetic intercorrelations. All 48 nodes, as pictured in Figure 6, exhibited Φ*k* = 0.58. Again, values >1.0 are suggestive of rich club properties. *E* in this network = 134 and *N* = 22, with a larger ratio of *E* to *N* being more indicative of rich club properties. In this network, increased edges stemmed from right central SA, right dorsomedial CT, and left orbitofrontal CT. Figure 7 presents the average nodal degree for each of the 48 parcellations, with yellow bars indicating those with degree >1 standard deviation from the mean.

It is possible that the small world properties could be an artifact of sparse connective properties across two different types of size measures, rather than within genetic CT- and SA-values. Therefore, we also separated parcellation-based CT- and SA-values and examined Φ*k* within each. CT and SA network mean degrees were 6.5 and 3.0, respectively. Given that the log of the number of nodes (24) is 3.17, only the CT network mean degree met this threshold, and only small world values for CT were estimable.

Within-SA connections exhibited no evidence of rich club properties (SA ρ_unc_*k* = 0.75, Φ*k* = 1.23, *E* = 72, *N* = 13), while within-CT did indicate possible rich club properties, such that the weighted ratio ρ_unc_*k* was > 1.0 (CT ρ_unc_*k* = 1.8, Φ*k* = 0.69, *E* = 45, *N* = 9). When parcellations are graphed such that edge length corresponds to strength of correlation, increased local genetic connections appear to surround right frontal SA and left medial CT parcellations (Figure 6).

## Discussion

This study used biometric modeling and graph theoretical approaches to examine emergent organizational principles from genetic associations among regional measures of brain size (both CT and SA), where the boundaries of the parcellations were determined *a priori* based on genetic relationships. Overall, results from these analyses suggest that (1) genetic covariances between structures in SA are less than in CT; (2) the genetic covariance between CT and SA for each structure is low; (3) the genetic covariance between SA and CT overall is low and basically negative; (4) the genetic correlations between homologous structures were the highest for SA and CT, and all positive.

Our finding of generally higher heritability for SA compared to CT measures is consistent with our previous findings within regions whose boundaries were determined based on sulcal/gyral anatomy (Kremen et al., 2010). Thus, using genetically-derived boundaries did not eliminate the apparent stronger contribution of genetic factors to SA vs. CT. Strong genetic associations between bilateral homologous regions are also consistent with earlier genetic research on bilateral brain structure in pediatric twins and siblings (Schmitt et al., 2008) and our own study in this sample using sulcal/gyral-based parcellations (Eyler et al., 2014). The current clustering findings illustrated in the dendrogram show some separation of SA from CT measures in terms of genetic correlations, which is consistent with previous results based on lobar parcellations (Panizzon et al., 2009) and based on the same parcellations used in the present analyses (Chen et al., 2012). However, by combining the two size measures (CT and SA) in a single correlation matrix, we were also able to examine whether unique regions in each hemisphere formed small distinct subunits of shared genetic covariance. It is possible that shared genetic covariance may reflect communalities in brain function or ontogeny.

A relatively dense local genetic network was observed between CT of orbitofrontal, superior temporal, precuneus, and dorsolateral prefrontal parcellations. Preliminary evidence of tight interconnected CT nodes, and sparse nodes of lower degree, indicate the possibility of a genetic CT network with rich club properties.

Graph theory has been applied to the brain in a wide variety of biological contexts, such as mapping of human brain circuitry and the human connectome (for a review, see Bullmore and Sporns, 2009; Filippi et al., 2013). Methods and tools to map networks continue to evolve (e.g., Cramer et al., 2010; Rubinov and Sporns, 2010; Borsboom et al., 2011; Borsboom and Cramer, 2013) and the use of graph theory has been especially fruitful for genetic imaging of development (Schmitt et al., 2008). Only Schmitt et al. have examined network properties emerging from genetic covariance matrices between brain regions. In that study, network properties were examined in genetic relationships between structural measures within anatomical brain regions in a pediatric sample. That study only focused on regional CT measures and not SA, and our results are consistent with that study, in that small world properties were observed in the genetic relationships between structurally-determined regions.

Notably, we found in the present study that SA was significantly negatively genetically associated with CT across a number of the connected parcellations. One possible explanation for negative genetic associations could be a competition or a “balancing out” between regions, e.g., for space within the physical confines of the skull, whose maximum size may be evolutionarily limited by the size of the birth canal. Another possibility is that cortical stretching might lead to a complex configuration of regional relationships between SA and CT. Globally, SA and CT have been observed to be genetically distinct (Panizzon et al., 2009; Vuoksimaa et al., 2015); however, our networks controlling for global measures of SA and CT suggest there are regional differences in the genetic correlations between SA and CT.

Research has identified unique rich club networks of the cortical connectome across species, and within human samples such networks have succeeded in differentiating healthy individuals from individuals with psychiatric disorders (van den Heuvel and Sporns, 2011; van den Heuvel et al., 2013; Collin et al., 2014). Our findings are from a healthy sample of individuals and do not say anything about psychiatric illness. However, results from this study suggest that future research could glean additional information from examination of genetic parcellation networks (particularly CT) in the context of aging and neurological disorders. One example would relate to whether genetic correlations between genetic parcellations are disparate across samples selected for family risk of disorder, or across healthy twins and twins discordant for disorder.

One caveat to these findings relates to the relatively small number of nodes examined. We chose 12 nodes in each hemisphere because that was the preferred number of clusters for both SA and CT in the original genetic parcellation study (Chen et al., 2012). However, genetic parcellation methods could be varied, in order to maximize the number of nodes or the regional shared genetic covariance between parcellations. Including size measures from a larger number of genetically-informative parcellations could help to further assess rich club and small world connectivity. Future research might also include joint analysis of subcortical measures with the cortical measures used here. However, even larger sample sizes would be needed to analyze more structures, and complications due to combining three different size measures (SA, CT, and volume) in the same statistical analysis may arise. Overall, future studies of genetically-informed imaging data would benefit from further characterization of genetic networks as potentially useful phenotypes for neuropsychological health and development.

## Author contributions

MT, WK, and CEF provided resources and coordination of data collection and processing. LM, LE, CF, and MP oversaw MRI processing and analyses. CC performed the genetic parcellation cluster analyses. AD and MN planned and undertook the statistical analyses, and MP and BV contributed to the biometrical analyses of the parcellation data. AD and CS conducted network analyses and AD drafted the first version of the manuscript. CS, MP, MN, LE, CF, CEF, CC, and WK contributed to subsequent drafts. All authors have reviewed and approved the final submission.

### Conflict of interest statement

The authors declare that the research was conducted in the absence of any commercial or financial relationships that could be construed as a potential conflict of interest.
