# Supplementary material for: Genetic network properties of the human cortex based on regional thickness and surface area measures
Source: Front Hum Neurosci. 2015 Aug 20;9:440. doi: 10.3389/fnhum.2015.00440 (PMC4542323; doi:10.3389/fnhum.2015.00440)
Supplement: Supplementary file 1 [file DataSheet1.DOCX]

Supplemental R Code for Univariate and Bivariate Twin Analyses

**# Univariate analyses of all of the clusters with confidence intervals**

require(OpenMx)

require(psych)

source("http://www.vipbg.vcu.edu/~vipbg/Tc24/GenEpiHelperFunctions.R")

twins <- read.csv("FOUNDATION adjusted clusters 9.25.14.csv",header=T)

#*** Summary statistics for the data file

describe(twins)

#*** Defining number of variables

tot <- dim(twins)[2]-4

tot #Should be number of variables to loop through

names(twins)[5:(4+tot)] #Variables to be used

## Creating MZ and DZ data sets ##

# Dividing up twins

twinA <- twins[twins$twin=="A",]

twinB <- twins[twins$twin=="B",]

# Remerging by case to create paired data set

newtwins <- merge(twinA, twinB, by=c("case","zyg14"),all.x=TRUE, all.y=TRUE,suffixes=c("_A","_B"))

names(newtwins) #Variable names in the now paired dataset

# Making data sets of Just MZ & DZ

MZdata <- as.data.frame(subset(newtwins,zyg14==1))

DZdata <- as.data.frame(subset(newtwins,zyg14==2))

# Renaming Data Before Starting Loop

MZdata1 <- MZdata

DZdata1 <- DZdata

#-----------------------------------------------------------------------#

### START OF LOOP ###

#-----------------------------------------------------------------------#

# Initializing Output

output <- c()

for (i in 1:tot){

## Defining Variables / Data for use with OpenMX ##

# Making Sparse Data Frame with only phenotype

MZdata <- MZdata1[,c(4+i,tot+6+i)]

DZdata <- DZdata1[,c(4+i,tot+6+i)]

# Getting Number of Twins Used in Analysis

cpMZ <- sum(complete.cases(MZdata)) # Number of complete MZ pairs

sMZ <- dim(MZdata)[1]-cpMZ # Number of MZ singletons

cpDZ <- sum(complete.cases(DZdata)) # Number of complete DZ pairs

sDZ <- dim(DZdata)[1]-cpDZ # Number of DZ singletons

# Define Variable to use in OpenMX

selvars <- c(names(MZdata))

Vars <- 'var'

nv <- 1 # Number of phenotypes

ntv <- nv*2

# Correlation between MZ twins

MZcor<-cor(MZdata,use="complete")

# Correlation between DZ twins

DZcor<-cor(DZdata,use="complete")

# Determining means and standard deviations of the data for use as start values later

totsd <- sd(na.omit(c(MZdata[,1],MZdata[,2],DZdata[,1],DZdata[,2])))

totmean <- mean(na.omit(c(MZdata[,1],MZdata[,2],DZdata[,1],DZdata[,2])))

#-----------------------------------------------------------------------#

### Univariate Analysis ###

## Fit Univariate Saturated Model ##

univTwinSatModel <- mxModel("univTwinSat",

mxModel("MZ",

mxMatrix( type="Lower", nrow=ntv, ncol=ntv, free=TRUE, values=totsd, name="CholMZ" ),

mxAlgebra( expression=CholMZ %*% t(CholMZ), name="expCovMZ" ),

mxMatrix( type="Full", nrow=1, ncol=ntv, free=TRUE, values=totmean, name="expMeanMZ" ),

mxData( observed=MZdata, type="raw" ),

mxFIMLObjective( covariance="expCovMZ", means="expMeanMZ", dimnames=selvars),

# Algebra's needed for equality constraints

mxAlgebra( expression=expMeanMZ[1,1:nv], name="expMeanMZt1"),

mxAlgebra( expression=expMeanMZ[1,(nv+1):ntv], name="expMeanMZt2"),

mxAlgebra( expression=t(diag2vec(expCovMZ)), name="expVarMZ"),

mxAlgebra( expression=expVarMZ[1,1:nv], name="expVarMZt1"),

mxAlgebra( expression=expVarMZ[1,(nv+1):ntv], name="expVarMZt2")

),

mxModel("DZ",

mxMatrix( type="Lower", nrow=ntv, ncol=ntv, free=TRUE, values=totsd, name="CholDZ" ),

mxAlgebra( expression=CholDZ %*% t(CholDZ), name="expCovDZ" ),

mxMatrix( type="Full", nrow=1, ncol=ntv, free=T, values=totmean, name="expMeanDZ" ),

mxData( observed=DZdata, type="raw" ),

mxFIMLObjective( covariance="expCovDZ", means="expMeanDZ", dimnames=selvars),

# Algebra's needed for equality constraints

mxAlgebra( expression=expMeanDZ[1,1:nv], name="expMeanDZt1"),

mxAlgebra( expression=expMeanDZ[1,(nv+1):ntv], name="expMeanDZt2"),

mxAlgebra( expression=t(diag2vec(expCovDZ)), name="expVarDZ"),

mxAlgebra( expression=expVarDZ[1,1:nv], name="expVarDZt1"),

mxAlgebra( expression=expVarDZ[1,(nv+1):ntv], name="expVarDZt2")

),

mxAlgebra( MZ.objective + DZ.objective, name="2sumll" ),

mxAlgebraObjective("2sumll") # Calculates the -2LL

)

univTwinSatFit <- mxRun(univTwinSatModel)

univTwinSatSumm <- summary(univTwinSatFit)

## Univariate ACE Model ##

twinACEModel <- mxModel("ACE",

# Matrices X, Y, and Z to store a, c, and e path coefficients

mxMatrix( type="Full", nrow=1, ncol=1, free=TRUE, values=totsd/3, label="a", name="X" ),

mxMatrix( type="Full", nrow=1, ncol=1, free=TRUE, values=totsd/3, label="c", name="Y" ),

mxMatrix( type="Full", nrow=1, ncol=1, free=TRUE, values=totsd/3, label="e", name="Z" ),

# Matrices A, C, and E compute variance components

mxAlgebra( expression=X %*% t(X), name="A" ),

mxAlgebra( expression=Y %*% t(Y), name="C" ),

mxAlgebra( expression=Z %*% t(Z), name="E" ),

mxAlgebra( expression=A+C+E, name="V" ),

mxAlgebra( expression=cbind(A/V,C/V,E/V),name="stndVCs"),

# Calculate 95% CIs here

mxCI(c("stndVCs")),

mxMatrix( type="Full", nrow=1, ncol=2, free=TRUE, values= totmean, label="mean", name="expMean" ),

# Algebra for expected variance/covariance matrix in MZ

mxAlgebra( expression= rbind ( cbind(A+C+E , A+C),

cbind(A+C , A+C+E)), name="expCovMZ" ),

# Algebra for expected variance/covariance matrix in DZ

mxAlgebra( expression= rbind ( cbind(A+C+E , 0.5%x%A+C),

cbind(0.5%x%A+C , A+C+E)), name="expCovDZ" ),

mxModel("MZ",

mxData( observed=MZdata, type="raw" ),

mxFIMLObjective( covariance="ACE.expCovMZ", means="ACE.expMean", dimnames=selvars ) ),

mxModel("DZ",

mxData( observed=DZdata, type="raw" ),

mxFIMLObjective( covariance="ACE.expCovDZ", means="ACE.expMean", dimnames=selvars ) ),

mxAlgebra( expression=MZ.objective + DZ.objective, name="twin" ),

mxAlgebraObjective("twin")

)

twinACEFit <- mxRun(twinACEModel,intervals=TRUE) # Additional indicator (interval=T) to calculate 95% CI must be included in mxRun statement

univACESumm <- summary(twinACEFit)

# Fitting AE model

twinAEModel <- mxRename(twinACEModel, "twinAE")

twinAEModel$twinAE.Y <- mxMatrix(type="Full",nrow=1,ncol=1,free=FALSE,values=0,label="c")

twinAEFit <- mxRun(twinAEModel,intervals=TRUE)

univAESumm <- summary(twinAEFit)

# Fitting CE model

twinCEModel <- mxRename(twinACEModel, "twinCE")

twinCEModel$twinCE.X <- mxMatrix(type="Full",nrow=1,ncol=1,free=FALSE,values=0,label="a")

twinCEFit <- mxRun(twinCEModel,intervals=TRUE)

univCESumm <- summary(twinCEFit)

# Fit E model

twinEModel <- mxRename(twinACEModel, "twinE")

twinEModel$twinE.X <- mxMatrix(type="Full",nrow=1,ncol=1,free=FALSE,values=0,label="a")

twinEModel$twinE.Y <- mxMatrix(type="Full",nrow=1,ncol=1,free=FALSE,values=0,label="c")

twinEFit <- mxRun(twinEModel)

univESumm <- summary(twinEFit)

#-----------------------------------------------------------------------#

## Generating the values that will go in the output file ##

varname <- names(twins)[4+i]

#Sat Model

sat2ll <- univTwinSatSumm$Minus2LogLikelihood

satdf <- univTwinSatSumm$degreesOfFreedom

#ACE

IFAIL<- twinACEFit@output$status[[1]]

ACEfit<- twinACEFit@output$Minus2LogLikelihood

AICace<- univACESumm$AIC

x2acefit<- ACEfit-sat2ll

pacefit<- pchisq(ACEfit-sat2ll,lower.tail=F,6)

#CE (test of no A)

CEfit<- twinCEFit@output$Minus2LogLikelihood

x2noA<- CEfit-ACEfit

pnoA<- pchisq(CEfit-ACEfit,lower.tail=F,1)

AICce<- univCESumm$AIC

#AE (test of no C)

AEfit<-twinAEFit@output$Minus2LogLikelihood

x2noC<- AEfit-ACEfit

pnoC<- pchisq(AEfit-ACEfit,lower.tail=F,1)

AICae<- univAESumm$AIC

#E Only (test of no A and C)

Efit<-twinEFit@output$Minus2LogLikelihood

x2noAC<- Efit-ACEfit

pnoAC<- pchisq(Efit-ACEfit,lower.tail=F,2)

AICe<- univESumm$AIC

#Expected Twin 1 Means

ExpMean1<-as.numeric(twinACEFit$ACE.expMean@values[1,1])

## Unstandardized ACE Parameter Estimates

#*** To change these, just change twinACEFit to best fit (i.e. twinCEFit)

a1<-twinACEFit@output$algebras$ACE.A

c1<-twinACEFit@output$algebras$ACE.C

e1<-twinACEFit@output$algebras$ACE.E

v1<-twinACEFit@output$algebras$ACE.V

## Standardized ACE Parameter Estimates

a2CI<-cbind(twinACEFit@output$algebras$ACE.stndVCs[1,1],twinACEFit@output$confidenceIntervals[1,1],twinACEFit@output$confidenceIntervals[1,2])

c2CI<-cbind(twinACEFit@output$algebras$ACE.stndVCs[1,2],twinACEFit@output$confidenceIntervals[2,1],twinACEFit@output$confidenceIntervals[2,2])

e2CI<-cbind(twinACEFit@output$algebras$ACE.stndVCs[1,3],twinACEFit@output$confidenceIntervals[3,1],twinACEFit@output$confidenceIntervals[3,2])

##Combine Output

full<-c(varname,MZcor[2,1],DZcor[2,1],IFAIL,ACEfit,x2acefit,pacefit,AICace,CEfit,x2noA,pnoA,AICce,

AEfit,x2noC,pnoC,AICae,Efit,x2noAC,pnoAC,AICe,ExpMean1,a1,c1,e1,v1,a2CI,c2CI,e2CI)

output <- rbind(output,full)}

### END OF LOOP ###

output <- as.data.frame(output,row.names=c(1:tot))

names(output)<-c("pheno","MZcor","DZcor","IFAIL","ACEfit","x2acefit","pacefit","AICace","CEfit","x2noA","pnoA","AICce",

"AEfit","x2noC","pnoC","AICae","Efit","x2noAC","pnoAC","AICe","ExpMean1","A","C","E","V",

"a2","a2Lower","a2Upper","c2","c2Lower","c2Upper","e2","e2Lower","e2Upper")

#Make an output file for use, will be created in directory specified at beginning

write.csv(output,"Cluster Univariate Output 9.25.14.csv",row.names=F)

************************************************************************************************************************************************

**# Bivariate AE analyses of all cluster pairs (multiple jobs)**

### Reading in Original Data ###

#*** Read in data file

twins <- read.csv("Parcellation_phenotypes_example.csv",header=T)

dim(twins)[1] # should be number of subjects

names(twins)[1:4] # should be subid, case, twin, zygosity

names(twins)[5] # should be primary phenotype, will be included in ALL analyses

names(twins[6:dim(twins)[2]]) # should be the remaining phenotypes

tot <- dim(twins)[2]-5

tot # should be number of variables to loop through

nv <- 2 #Number of phenotypes (NVAR)

ntv <- nv*2

## Creating MZ and DZ data sets ##

# Dividing up twins

twinA <- twins[twins$twin=="A",]

twinB <- twins[twins$twin=="B",]

# Remerging by case to create paired data set

newtwins <- merge(twinA, twinB, by=c("case","zyg14"),all.x=TRUE, all.y=TRUE,suffixes=c("_A","_B"))

# Making data sets of Just MZ & DZ

MZdata <- as.data.frame(subset(newtwins,zyg14==1))

DZdata <- as.data.frame(subset(newtwins,zyg14==2))

# Renaming Data Before Starting Loop

MZdata1 <- MZdata

DZdata1 <- DZdata

#-----------------------------------------------------------------------#

### START OF LOOP ###

#-----------------------------------------------------------------------#

# Initializing Output

output <- c()

for (k in 1:tot){

## Defining Variables / Data for use with OpenMX ##

# Making Sparse Data Frame with only phenotype

MZdata <- MZdata1[,c(5,5+k,tot+8,tot+8+k)]

DZdata <- DZdata1[,c(5,5+k,tot+8,tot+8+k)]

# Getting Number of Twins Used in Analysis

cpMZ <- sum(complete.cases(MZdata)) # Number of complete MZ pairs

sMZ <- dim(MZdata)[1]-cpMZ # Number of MZ singletons

cpDZ <- sum(complete.cases(DZdata)) # Number of complete DZ pairs

sDZ <- dim(DZdata)[1]-cpDZ # Number of DZ singletons

# Define Variable to use in OpenMX

selVars <- c(names(MZdata))

# Getting Number of Twins Used in Analysis

cpMZ <- sum(complete.cases(MZdata)) # Number of complete MZ pairs

sMZ <- dim(MZdata)[1]-cpMZ # Number of MZ singletons

cpDZ <- sum(complete.cases(DZdata)) # Number of complete DZ pairs

sDZ <- dim(DZdata)[1]-cpDZ # Number of DZ singletons

totsd <- sd(na.omit(c(MZdata[,1],MZdata[,2],DZdata[,1],DZdata[,2])))

totmean <- mean(na.omit(c(MZdata[,1],MZdata[,2],DZdata[,1],DZdata[,2])))

pheno1 <- as.character((names(twins[5])))

pheno2 <- as.character((names(twins[5+k])))

print(pheno2)

# Example Code: Fit Bivariate Saturated Model

# -----------------------------------------------------------------------

meanSV <- c(5,5) # Start values for the means

cholSV <- c(.9,0,0,0,.9,0,0,.9,0,.9) # Start values for the variance/covariance matrix

repM <- rep("M",10)

repD <- rep("D",10)

row <- c(1,2,3,4,2,3,4,3,4,4)

col <- c(1,1,1,1,2,2,2,3,3,4)

labM <- paste(repM,row,col,sep="")

meanM <- paste("meanM",c(1,2),c("A","A","B","B"),sep="")

meanD <- paste("meanD",c(1,2),c("A","A","B","B"),sep="")

labD <- paste(repD,row,col,sep="")

multiTwinSatModel <- mxModel("multiTwinSat",

mxModel("MZ",

mxMatrix( type="Lower", nrow=ntv, ncol=ntv, free=TRUE, values=cholSV, labels=labM, name="CholMZ" ),

mxAlgebra( expression=CholMZ %*% t(CholMZ), name="expCovMZ" ),

mxMatrix( type="Full", nrow=1, ncol=ntv, free=TRUE, values=meanSV, labels=meanM, name="expMeanMZ" ),

mxData( observed=MZdata, type="raw" ),

mxFIMLObjective( covariance="expCovMZ", means="expMeanMZ", dimnames=selVars),

# Algebra's needed for equality constraints

mxAlgebra( expression=t(diag2vec(expCovMZ)), name="expVarMZ"),

mxAlgebra( expression=expVarMZ[1,1:nv], name="expVarMZtA"),

mxAlgebra( expression=expVarMZ[1,(nv+1):ntv], name="expVarMZtB"),

mxAlgebra( expression=expMeanMZ[1,1:nv], name="expMeanMZtA"),

mxAlgebra( expression=expMeanMZ[1,(nv+1):ntv], name="expMeanMZtB")

),

mxModel("DZ",

mxMatrix( type="Lower", nrow=ntv, ncol=ntv, free=TRUE, values=cholSV, labels=labD, name="CholDZ" ),

mxAlgebra( expression=CholDZ %*% t(CholDZ), name="expCovDZ" ),

mxMatrix( type="Full", nrow=1, ncol=ntv, free=TRUE, values=meanSV, labels=meanD, name="expMeanDZ" ),

mxData( observed=DZdata, type="raw" ),

mxFIMLObjective( covariance="expCovDZ", means="expMeanDZ", dimnames=selVars),

# Algebra's needed for equality constraints

mxAlgebra( expression=t(diag2vec(expCovDZ)), name="expVarDZ"),

mxAlgebra( expression=expVarDZ[1,1:nv], name="expVarDZtA"),

mxAlgebra( expression=expVarDZ[1,(nv+1):ntv], name="expVarDZtB"),

mxAlgebra( expression=expMeanDZ[1,1:nv], name="expMeanDZtA"),

mxAlgebra( expression=expMeanDZ[1,(nv+1):ntv], name="expMeanDZtB")

),

mxAlgebra( MZ.objective + DZ.objective, name="neg2sumll" ),

mxAlgebraObjective("neg2sumll")

)

multiTwinSatFit <- mxRun(multiTwinSatModel)

multiTwinSatSumm <- summary(multiTwinSatFit)

#multiTwinSatSumm

# Generate Saturated Output

#parameterSpecifications(multiTwinSatFit)

#expectedMeansCovariances(multiTwinSatFit)

#tableFitStatistics(multiTwinSatFit)

# Fit Bivariate ACE Model with Raw Data Input

# -----------------------------------------------------------------------

meanSVnv <- rep(5,nv) # Start values for the means

cholASVnv <- rep(.3,nv*(nv+1)/2) # Start values for the genetic and environmental parameter estimates

cholCSVnv <- rep(.1,nv*(nv+1)/2)

cholESVnv <- rep(.3,nv*(nv+1)/2)

rows <- c()

cols <- c()

for (i in 1:nv){

row <- c(i:nv)

col <- rep(i,(nv+1-i))

rows <- c(rows,row)

cols <- c(cols,col)

}

AFac <- paste("A",rows,cols,sep="")

CFac <- paste("C",rows,cols,sep="")

EFac <- paste("E",rows,cols,sep="")

mean <- as.character(c("mean1","mean2"))

multiCholACEModel <- mxModel("ACE",

# Matrices a, c, and e to store a, c, and e path coefficients

mxMatrix( type="Lower", nrow=nv, ncol=nv, free=TRUE, values=cholASVnv, labels=AFac, name="a" ),

mxMatrix( type="Lower", nrow=nv, ncol=nv, free=TRUE, values=cholCSVnv, labels=CFac, name="c" ),

mxMatrix( type="Lower", nrow=nv, ncol=nv, free=TRUE, values=cholESVnv, labels=EFac, name="e" ),

# Matrices A, C, and E compute variance components

mxAlgebra( expression=a %*% t(a), name="A" ),

mxAlgebra( expression=c %*% t(c), name="C" ),

mxAlgebra( expression=e %*% t(e), name="E" ),

# Algebra to compute total variances and standard deviations (diagonal only)

mxAlgebra( expression=A+C+E, name="V" ),

mxMatrix( type="Iden", nrow=nv, ncol=nv, name="I"),

mxAlgebra( expression=solve(sqrt(I*V)), name="iSD"),

# Confidence intervals portion for covariance matrices

mxAlgebra( expression=A/V,name="stndVCA"),

mxAlgebra( expression=C/V,name="stndVCC"),

mxAlgebra( expression=E/V,name="stndVCE"),

mxCI(c("stndVCA","stndVCC","stndVCE")),

# Confidence intervals for Phenotypic correlation pieces

mxAlgebra((solve(sqrt(I*V)) %*% V %*% solve(sqrt(I*V)))[1,2],name="Vcorr"),

mxAlgebra((solve(sqrt(I*A)) %*% A %*% solve(sqrt(I*A)))[1,2],name="Acorr"),

mxAlgebra((solve(sqrt(I*C)) %*% C %*% solve(sqrt(I*C)))[1,2],name="Ccorr"),

mxAlgebra((solve(sqrt(I*E)) %*% E %*% solve(sqrt(I*E)))[1,2],name="Ecorr"),

mxCI(c("Vcorr","Acorr","Ccorr","Ecorr")),

## Note that the rest of the mxModel statements do not change for bi/multivariate case

# Matrix & Algebra for expected means vector

mxMatrix( type="Full", nrow=1, ncol=nv, free=TRUE, values=meanSVnv, labels=mean, name="Mean" ),

mxAlgebra( expression= cbind(Mean,Mean), name="expMean"),

# Algebra for expected variance/covariance matrix in MZ

mxAlgebra( expression= rbind ( cbind(A+C+E , A+C),

cbind(A+C , A+C+E)), name="expCovMZ" ),

# Algebra for expected variance/covariance matrix in DZ

mxAlgebra( expression= rbind ( cbind(A+C+E , 0.5%x%A+C),

cbind(0.5%x%A+C , A+C+E)), name="expCovDZ" ),

mxModel("MZ",

mxData( observed=MZdata, type="raw" ),

mxFIMLObjective( covariance="ACE.expCovMZ", means="ACE.expMean", dimnames=selVars )

),

mxModel("DZ",

mxData( observed=DZdata, type="raw" ),

mxFIMLObjective( covariance="ACE.expCovDZ", means="ACE.expMean", dimnames=selVars )

),

mxAlgebra( expression=MZ.objective + DZ.objective, name="neg2sumll" ),

mxAlgebraObjective("neg2sumll")

)

multiCholACEFit <- mxRun(multiCholACEModel,intervals=TRUE) # Allows for confidence intervals to be calculated

multiCholACESumm <- summary(multiCholACEFit)

multiCholACEFit

GenCorr <- multiCholAEFit@output$algebras$AE.Acorr

# Generate Bivariate Cholesky ACE Output

#parameterSpecifications(multiCholACEFit)

#expectedMeansCovariances(multiCholACEFit)

#tableFitStatistics(multiTwinSatFit, multiCholACEFit)

# Generate List of Parameter Estimates and Derived Quantities using formatOutputMatrices

# Set r_C = 0

# -----------------------------------------------------------------------

multiCholACEModel_noCcorr <- mxRename(multiCholACEModel, "ACE")

multiCholACEModel_noCcorr$ACE.c <- mxMatrix( type="Lower", nrow=nv, ncol=nv, free=c(TRUE,FALSE,TRUE), values=c(.1,0,.1), labels=CFac, name="c" )

multiCholACEFit_noCcorr <- mxRun(multiCholACEModel_noCcorr)

multiCholACESumm_noCcorr <- summary(multiCholACEFit_noCcorr)

#multiCholACESumm_noCcorr

#tableFitStatistics(multiCholACEFit,multiCholACEFit_noCcorr)

# Cholesky Output (Path Estimates)

#formatOutputMatrices(multiCholACEFit_noCcorr,ACEpathMatrices,ACEpathLabels,Vars,4)

# Correlated Factors Output (Cov and Corr Matrices)

#formatOutputMatrices(multiCholACEFit_noCcorr,ACEcovMatrices,ACEcovLabels,Vars,4)

# Set r_A = 0

# -----------------------------------------------------------------------

multiCholACEModel_noAcorr <- mxRename(multiCholACEModel, "ACE")

multiCholACEModel_noAcorr$ACE.a <- mxMatrix( type="Lower", nrow=nv, ncol=nv, free=c(TRUE,FALSE,TRUE), values=c(.3,0,.3), labels=AFac, name="a")

multiCholACEFit_noAcorr <- mxRun(multiCholACEModel_noAcorr)

multiCholACESumm_noAcorr <- summary(multiCholACEFit_noAcorr)

#multiCholACESumm_noAcorr

#tableFitStatistics(multiCholACEFit,multiCholACEFit_noAcorr)

# Cholesky Output (Path Estimates)

#formatOutputMatrices(multiCholACEFit_noAcorr,ACEpathMatrices,ACEpathLabels,Vars,4)

# Correlated Factors Output (Cov and Corr Matrices)

#formatOutputMatrices(multiCholACEFit_noAcorr,ACEcovMatrices,ACEcovLabels,Vars,4)

# Set r_E = 0

# -----------------------------------------------------------------------

multiCholACEModel_noEcorr <- mxRename(multiCholACEModel, "ACE")

multiCholACEModel_noEcorr$ACE.e <- mxMatrix( type="Lower", nrow=nv, ncol=nv, free=c(TRUE,FALSE,TRUE), values=c(.3,0,.3), labels=EFac, name="e")

multiCholACEFit_noEcorr <- mxRun(multiCholACEModel_noEcorr)

multiCholACESumm_noEcorr <- summary(multiCholACEFit_noEcorr)

#multiCholACESumm_noEcorr

#tableFitStatistics(multiCholACEFit,multiCholACEFit_noEcorr)

# Cholesky Output (Path Estimates)

#formatOutputMatrices(multiCholACEFit_noEcorr,ACEpathMatrices,ACEpathLabels,Vars,4)

# Correlated Factors Output (Cov and Corr Matrices)

#formatOutputMatrices(multiCholACEFit_noEcorr,ACEcovMatrices,ACEcovLabels,Vars,4)

# Set r_A = 0 & r_C = 0

# -----------------------------------------------------------------------

multiCholACEModel_noACcorr <- mxRename(multiCholACEModel, "ACE")

multiCholACEModel_noACcorr$ACE.a <- mxMatrix( type="Lower", nrow=nv, ncol=nv, free=c(TRUE,FALSE,TRUE), values=c(.3,0,.3), labels=AFac, name="a")

multiCholACEModel_noACcorr$ACE.c <- mxMatrix( type="Lower", nrow=nv, ncol=nv, free=c(TRUE,FALSE,TRUE), values=c(.1,0,.1), labels=CFac, name="c")

multiCholACEFit_noACcorr <- mxRun(multiCholACEModel_noACcorr)

multiCholACESumm_noACcorr <- summary(multiCholACEFit_noACcorr)

#multiCholACESumm_noACcorr

#tableFitStatistics(multiCholACEFit,multiCholACEFit_noACcorr)

# Cholesky Output (Path Estimates)

#formatOutputMatrices(multiCholACEFit_noACcorr,ACEpathMatrices,ACEpathLabels,Vars,4)

# Correlated Factors Output (Cov and Corr Matrices)

#formatOutputMatrices(multiCholACEFit_noACcorr,ACEcovMatrices,ACEcovLabels,Vars,4)

# Set r_A = 0 & r_E = 0

# -----------------------------------------------------------------------

multiCholACEModel_noAEcorr <- mxRename(multiCholACEModel, "ACE")

multiCholACEModel_noAEcorr$ACE.a <- mxMatrix( type="Lower", nrow=nv, ncol=nv, free=c(TRUE,FALSE,TRUE), values=c(.3,0,.3), labels=AFac, name="a")

multiCholACEModel_noAEcorr$ACE.e <- mxMatrix( type="Lower", nrow=nv, ncol=nv, free=c(TRUE,FALSE,TRUE), values=c(.3,0,.3), labels=EFac, name="e")

multiCholACEFit_noAEcorr <- mxRun(multiCholACEModel_noACcorr)

multiCholACESumm_noAEcorr <- summary(multiCholACEFit_noACcorr)

#multiCholACESumm_noAEcorr

#tableFitStatistics(multiCholACEFit,multiCholACEFit_noAEcorr)

# Cholesky Output (Path Estimates)

#formatOutputMatrices(multiCholACEFit_noAEcorr,ACEpathMatrices,ACEpathLabels,Vars,4)

# Correlated Factors Output (Cov and Corr Matrices)

#formatOutputMatrices(multiCholACEFit_noACcorr,ACEcovMatrices,ACEcovLabels,Vars,4)

# Set r_E = 0 & r_C = 0

# -----------------------------------------------------------------------

multiCholACEModel_noCEcorr <- mxRename(multiCholACEModel, "ACE")

multiCholACEModel_noCEcorr$ACE.e <- mxMatrix( type="Lower", nrow=nv, ncol=nv, free=c(TRUE,FALSE,TRUE), values=c(.3,0,.3), labels=EFac, name="e")

multiCholACEModel_noCEcorr$ACE.c <- mxMatrix( type="Lower", nrow=nv, ncol=nv, free=c(TRUE,FALSE,TRUE), values=c(.1,0,.1), labels=CFac, name="c")

multiCholACEFit_noCEcorr <- mxRun(multiCholACEModel_noCEcorr)

multiCholACESumm_noCEcorr <- summary(multiCholACEFit_noCEcorr)

#multiCholACESumm_noCEcorr

#tableFitStatistics(multiCholACEFit,multiCholACEFit_noCEcorr)

# Cholesky Output (Path Estimates)

#formatOutputMatrices(multiCholACEFit_noCEcorr,ACEpathMatrices,ACEpathLabels,Vars,4)

# Correlated Factors Output (Cov and Corr Matrices)

#formatOutputMatrices(multiCholACEFit_noCEcorr,ACEcovMatrices,ACEcovLabels,Vars,4)

# Test overall phenotypic correlation = 0 (i.e, r_A = r_C = r_E = 0)

# -----------------------------------------------------------------------

multiCholACEModel_nocorr <- mxRename(multiCholACEModel, "ACE")

multiCholACEModel_nocorr$ACE.a <- mxMatrix( type="Lower", nrow=nv, ncol=nv, free=c(TRUE,FALSE,TRUE), values=c(.3,0,.3), labels=AFac, name="a")

multiCholACEModel_nocorr$ACE.c <- mxMatrix( type="Lower", nrow=nv, ncol=nv, free=c(TRUE,FALSE,TRUE), values=c(.1,0,.1), labels=CFac, name="c")

multiCholACEModel_nocorr$ACE.e <- mxMatrix( type="Lower", nrow=nv, ncol=nv, free=c(TRUE,FALSE,TRUE), values=c(.3,0,.3), labels=EFac, name="e")

multiCholACEFit_nocorr <- mxRun(multiCholACEModel_nocorr)

multiCholACESumm_nocorr <- summary(multiCholACEFit_nocorr)

#multiCholACESumm_nocorr

#tableFitStatistics(multiCholACEFit,multiCholACEFit_nocorr)

# Cholesky Output (Path Estimates)

#formatOutputMatrices(multiCholACEFit_nocorr,ACEpathMatrices,ACEpathLabels,Vars,4)

# Correlated Factors Output (Cov and Corr Matrices)

#formatOutputMatrices(multiCholACEFit_nocorr,ACEcovMatrices,ACEcovLabels,Vars,4)

# Set C = 0, Bivariate AE Model

# -----------------------------------------------------------------------

multiCholAEModel <- mxRename(multiCholACEModel, "AE")

multiCholAEModel$AE.c <- mxMatrix( type="Full", nrow=nv, ncol=nv, free=FALSE, values=0, name="c" )

multiCholAEModel$AE.Ccorr <- mxAlgebra(0,name="Ccorr")

multiCholAEFit <- mxRun(multiCholAEModel,intervals=T)

multiCholAESumm <- summary(multiCholAEFit)

#multiCholAESumm

#tableFitStatistics(multiCholACEFit,multiCholAEFit)

# Cholesky Output (Path Estimates)

AEpathMatrices <- c("AE.a","AE.c","AE.e","AE.iSD","AE.iSD %*% AE.a","AE.iSD %*% AE.c","AE.iSD %*% AE.e")

#formatOutputMatrices(multiCholAEFit,AEpathMatrices,ACEpathLabels,Vars,4)

# Correlated Factors Output (Cov and Corr Matrices)

AEcovMatrices <- c("AE.A","AE.C","AE.E","AE.V","AE.A/AE.V","AE.C/AE.V","AE.E/AE.V")

#formatOutputMatrices(multiCholAEFit,AEcovMatrices,ACEcovLabels,Vars,4)

#-------------------------------------------------------------------#

### OUTPUT ###

#-------------------------------------------------------------------#

sat2ll <- multiTwinSatSumm$Minus2LogLikelihood

satdf <- multiTwinSatFit$degreesOfFreedom

#ACE

IFAIL<- multiCholACEFit@output$status[[1]]

ACEfit<- multiCholACEFit@output$Minus2LogLikelihood

AICace<- multiCholACESumm$AIC

x2acefit<- ACEfit-sat2ll

pacefit<- pchisq(ACEfit-sat2ll,lower.tail=F,17)

# Path Estimates, Standardized

sd <- multiCholACEFit@output$algebra$ACE.iSD

a <- multiCholACEFit@output$matrices$ACE.a

c <- multiCholACEFit@output$matrices$ACE.c

e <- multiCholACEFit@output$matrices$ACE.e

Apath <- sd %*% a

Cpath <- sd %*% c

Epath <- sd %*% e

LP1_pheno1_A <- Apath[1,1]

LP1_pheno2_A <- Apath[2,1]

LP2_pheno2_A <- Apath[2,2]

LP1_pheno1_C <- Cpath[1,1]

LP1_pheno2_C <- Cpath[2,1]

LP2_pheno2_C <- Cpath[2,2]

LP1_pheno1_E <- Epath[1,1]

LP1_pheno2_E <- Epath[2,1]

LP2_pheno2_E <- Epath[2,2]

# Correlated Factors Output (Cov and Corr Matrices), Standardized

A <- multiCholACEFit@output$algebra$ACE.A

C <- multiCholACEFit@output$algebra$ACE.C

E <- multiCholACEFit@output$algebra$ACE.E

V <- multiCholACEFit@output$algebra$ACE.V

Acov <- A/V

Ccov <- C/V

Ecov <- E/V

A1 <- Acov[1,1]

A2 <- Acov[2,2]

A12 <- Acov[1,2]

C1 <- Ccov[1,1]

C2 <- Ccov[2,2]

C12 <- Ccov[1,2]

E1 <- Ecov[1,1]

E2 <- Ecov[2,2]

E12 <- Ecov[1,2]

# Test of no A corr

ACEfit_noAcorr<- multiCholACEFit_noAcorr@output$Minus2LogLikelihood

x2noAcorr<- ACEfit_noAcorr-ACEfit

pnoAcorr<- pchisq(ACEfit_noAcorr-ACEfit,lower.tail=F,1)

AICace_noAcorr<- multiCholACESumm_noAcorr$AIC

# Test of no C corr

ACEfit_noCcorr<-multiCholACEFit_noCcorr@output$Minus2LogLikelihood

x2noCcorr<- ACEfit_noCcorr-ACEfit

pnoCcorr<- pchisq(ACEfit_noCcorr-ACEfit,lower.tail=F,1)

AICace_noCcorr<- multiCholACESumm_noCcorr$AIC

#AC (test of no E corr)

ACEfit_noEcorr<-multiCholACEFit_noEcorr@output$Minus2LogLikelihood

x2noEcorr<- ACEfit_noEcorr-ACEfit

pnoEcorr<- pchisq(ACEfit_noEcorr-ACEfit,lower.tail=F,1)

AICace_noEcorr<- multiCholACESumm_noEcorr$AIC

#E Only (test of no A or C correlation)

ACEfit_noACcorr<-multiCholACEFit_noACcorr@output$Minus2LogLikelihood

x2noACcorr<- ACEfit_noACcorr-ACEfit

pnoACcorr<- pchisq(ACEfit_noACcorr-ACEfit,lower.tail=F,2)

AICace_noACcorr<- multiCholACESumm_noACcorr$AIC

#C Only (test of no A or E correlation)

ACEfit_noAEcorr<-multiCholACEFit_noAEcorr@output$Minus2LogLikelihood

x2noAEcorr<- ACEfit_noAEcorr-ACEfit

pnoAEcorr<- pchisq(ACEfit_noAEcorr-ACEfit,lower.tail=F,2)

AICace_noAEcorr<- multiCholACESumm_noAEcorr$AIC

#A Only (test of no C or E correlation)

ACEfit_noCEcorr<-multiCholACEFit_noCEcorr@output$Minus2LogLikelihood

x2noCEcorr<- ACEfit_noCEcorr-ACEfit

pnoCEcorr<- pchisq(ACEfit_noCEcorr-ACEfit,lower.tail=F,2)

AICace_noCEcorr<- multiCholACESumm_noCEcorr$AIC

#No Phenotypic Corr

ACEfit_nocorr<-multiCholACEFit_nocorr@output$Minus2LogLikelihood

x2nocorr<- ACEfit_nocorr-ACEfit

pnocorr<- pchisq(ACEfit_nocorr-ACEfit,lower.tail=F,2)

AICace_nocorr<- multiCholACESumm_nocorr$AIC

##Combine Output

bivariate<-c(pheno1,pheno2,sat2ll,ACEfit,x2acefit,pacefit,GenCorr, AICace,

LP1_pheno1_A, LP1_pheno2_A, LP2_pheno2_A, LP1_pheno1_C, LP1_pheno2_C, LP2_pheno2_C, LP1_pheno1_E, LP1_pheno2_E, LP2_pheno2_E,

A1, A12, A2, C1, C12, C2, E1, E12, E2,

ACEfit_noAcorr,x2noAcorr,pnoAcorr,AICace_noAcorr,

ACEfit_noCcorr,x2noCcorr,pnoCcorr,AICace_noCcorr,ACEfit_noEcorr,x2noEcorr,pnoEcorr,AICace_noEcorr,

ACEfit_noACcorr,x2noACcorr,pnoACcorr,AICace_noACcorr,

ACEfit_noAEcorr,x2noAEcorr,pnoAEcorr,AICace_noAEcorr,

ACEfit_noCEcorr,x2noCEcorr,pnoCEcorr,AICace_noCEcorr,

ACEfit_nocorr,x2nocorr,pnocorr,AICace_nocorr)

output <- rbind(output, bivariate)

}

## END OF LOOP ##

output <- as.data.frame(output, row.names = c(1:tot))

names(output)<-c("variable1","variable2","satfit","ACEfit","x2acefit","pacefit","GenCorr","AICace",

"stpathLP1_pheno1_A","stpathLP1_pheno2_A","stpathLP2_pheno2_A",

"stpathLP1_pheno1_C","stpathLP1_pheno2_C","stpathLP2_pheno2_C",

"stpathLP1_pheno1_E","stpathLP1_pheno2_E","stpathLP2_pheno2_E",

"stCovA11", "stCovA12", "stCovA22", "stCovC11", "stCovC12", "stCovC22", "stCovE11", "stCovE12", "stCovE22",

"ACEfit_noAcorr","x2noAcorr","pnoAcorr","AICace_noAcorr",

"ACEfit_noCcorr","x2noCcorr","pnoCcorr","AICace_noCcorr",

"ACEfit_noEcorr","x2noEcorr","pnoEcorr","AICeace_noEcorr",

"ACEfit_noACcorr","x2noACcorr","pnoACcorr","AICace_noACcorr",

"ACEfit_noAEcorr","x2noAEcorr","pnoAEcorr","AICace_noAEcorr",

"ACEfit_noCEcorr","x2noCEcorr","pnoCEcorr","AICeace_noCEcorr",

"ACEfit_nocorr","x2nocorr","pnocorr","AICace_nocorr")

write.csv(output, "Example_bivariates_1.csv", row.names=F)
