# Supplementary figures and images for: Genetic network properties of the human cortex based on regional thickness and surface area measures
Source: Front Hum Neurosci. 2015 Aug 20;9:440. doi: 10.3389/fnhum.2015.00440 (PMC4542323; doi:10.3389/fnhum.2015.00440)

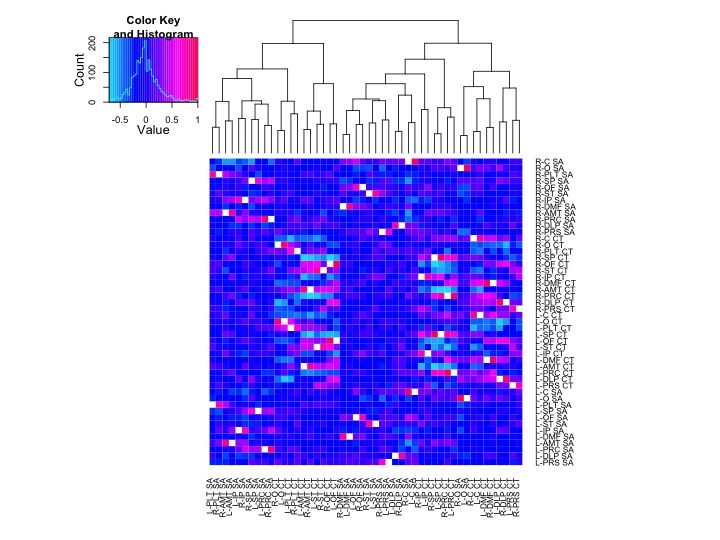

Supplement: Supplementary file 2 [file Image1.JPEG]
